# Peer Review of “Evaluating Population Density as a Parameter for Optimizing COVID-19 Testing: Statistical Analysis”

**DOI:** 10.2196/27103

**Published:** 2021-02-03

**Keywords:** infectious diseases, testing, population density, policy, coronavirus, COVID-19, SARS-CoV-2

*This is a peer review submitted for the paper “Evaluating Population Density as a Parameter for Optimizing COVID-19 Testing: Statistical Analysis.”*

## Round 1 Review

### General Comments

This paper [1] signals the need for a more nuanced COVID-19 testing strategy. The authors propose using population density–driven testing to help address this need. Testing strategies certainly have room for improvement and continuous assessment, especially in emergent situations like COVID-19. Maps are great visualization tools.

### Specific Comments

#### Major Comments

This paper communicates that adjusting testing strategies by population density will save lives and livelihoods. While I think there is merit to finding effective ways to account for population density, especially in contexts with high-quality census and robust public health surveillance data, there is a host of other dynamic factors that play into the complicated pathway between population density, testing, and saving lives and livelihoods that are not accounted for in the current version of this paper.

This draft also uses absolute terms and expressions that do not seem appropriate given the scope of the study. The authors might benefit from speaking in less absolute terms, remove anecdotal examples such as the elevator vs football field in exchange for more standardized epidemiological measures, and include in the paper a discussion about the limitations of using their proposed population density–driven testing. The paper should also speak more to the nature (eg, challenges) of public health data, monitoring and surveillance, and the role of testing in this context. As a policy-oriented paper, it should also discuss more of the potential impacts of modifying a testing strategy (pros and cons), including the costs associated with changing the current testing strategy. The paper might also want to address whether or not adjusted testing strategies based on population density (or similar measures) have successfully been done elsewhere.
